# The Analysis of Food Intake in Patients with Cirrhosis Waiting for Liver Transplantation: A Neglected Step in the Nutritional Assessment

**DOI:** 10.3390/nu11102462

**Published:** 2019-10-15

**Authors:** Francesco Palmese, Ilaria Bolondi, Ferdinando Antonino Giannone, Giacomo Zaccherini, Manuel Tufoni, Maurizio Baldassarre, Paolo Caraceni

**Affiliations:** 1 Department of Medical and Surgical Sciences, Alma Mater Studiorum University of Bologna, 40138 Bologna, Italy; palmesefrancesco@gmail.com (F.P.); ilaria_bolondi@yahoo.it (I.B.); g.zaccherini@gmail.com (G.Z.); manuel.tufoni@gmail.com (M.T.); baldassarre.maurizio@gmail.com (M.B.); 2 Support Services and People Care, General Direction, S. Orsola-Malpighi University Hospital, 40138 Bologna, Italy; ferdinando.giannone@gmail.com; 3 Centre for Applied Biomedical Research (C.R.B.A.), Alma Mater Studiorum University of Bologna, 40138 Bologna, Italy

**Keywords:** malnutrition, cirrhosis, liver transplantation, food records, caloric intake, macronutrients intakes, patient adherence

## Abstract

Patients with cirrhosis waiting for liver transplantation (LT) frequently present a nutritional disorder, which represents an independent predictor of morbidity and mortality before and after transplantation. Thus, a proper assessment of the food intake by using different methods, such as food records, food frequency questionnaires, and 24 h recall, should be deemed an important step of the nutritional management of these patients. The available published studies indicate that the daily food intake is inadequate in the majority of waitlisted patients. These findings were confirmed by our experience, showing that the daily intake of total calories, proteins and carbohydrates was inadequate in approximately 85–95% of patients, while that of lipids and simple carbohydrates was inadequate in almost 50% of them. These data highlight the need to implement an effective educational program provided by certified nutritionists or dieticians, who should work in close collaboration with the hepatologist to provide a nutritional intervention tailored to the individual patient requirements.

## 1. Introduction

Patients with end-stage cirrhosis waiting for liver transplantation (LT) are frequently malnourished, showing different nutritional disorders, e.g., undernutrition, sarcopenia and obesity, which may also coexist in the same patient [1,2]. Besides the intuitive association between undernutrition and sarcopenia, the prevalence of sarcopenic obesity is increasing over the last years due to the growing number of patients with cirrhosis caused by non-alcoholic steatohepatitis (NASH) [3,4,5,6]. Although a wide variability of diagnostic methods used, among studies the prevalence of nutritional disorders results usually high (up to 75%) [2]. Instead, there is a worldwide accepted consensus that malnutrition represents an independent predictor of morbidity and mortality in patients with cirrhosis before and after LT [1,7].

In a systematic review, it has been observed that both severe under nutrition (BMI < 18.5 kg/m^2^) and severe obesity (BMI > 40 kg/m^2^) in patients undergoing liver transplantation are associated with increased mortality and complications [8]. Obesity, diabetes and familiarity for diabetes are independent risk factors for the development of metabolic syndrome after liver transplantation, which is associated with a higher risk of cardiovascular complications, malignancy and poor outcome [9,10]. On the other hand, patients with pre-operative malnutrition undergoing liver transplantation have higher mortality, susceptibility to infections, longer hospital stay (both intensive care unit and regular ward) after liver transplantation [11,12]. Moreover, very low protein intake in patients awaiting liver transplantation is associated with malnutrition and mortality [13]. There are no conclusive formal trials showing that preoperative nutritional intervention improves clinical outcome [1], but it is not surprising that in recent years, a growing emphasis has been placed on the importance of nutrition in the global management of patients with cirrhosis and, more specifically, in candidates for LT. To this effect, recommendations regarding the amount, type and distribution of dietary macro- and micro-nutrients as well as the assessment and diagnosis of nutritional disorders in patients with cirrhosis have been recently published by several international and national scientific societies [1,14,15]. A brief general synthesis of these indications is shown in Table 1.

As a result, many transplant hepatologists are currently including a screening for nutritional disorders as a part of their regular clinical work-up. Among the screening tools for the risk of malnutrition, the Royal Free Hospital—Global Assessment and the Royal Free Hospital—Prioritizing Tool have been validated in patients with cirrhosis [16]. Furthermore, due to its clearly established strong negative impact on patient’s frailty and pre- and post-transplant outcomes [17], the use of screening tools for sarcopenia (i.e., hand-grip strength test, 4 m walking test, chair standing test, 6 min walking test, etc.) and its objective quantification with imaging techniques (computed tomography (CT) or magnetic resonance (MR)) is spreading in clinical practice [17].

Once a nutritional disorder or at least a condition of high risk has been demonstrated, the analysis of the current dietary habits would be particularly useful in order to unveil the discrepancies with the established nutritional recommendations and to effectively improve patient adherence to them. Unfortunately, this step is often neglected.

## 2. The Assessment of Food Intake

Several tools have been developed to assess food intake, including the Food Records (FRs), the Food Frequency Questionnaire (FFQ), and the 24 h recall (24 hR) [18]. There is still an open debate on which tool can be considered the most accurate due to the limitations related to misreporting, technical reproducibility and the availability of resources [19,20,21,22]. Main advantages and disadvantages of each tool are resumed in Table 2.

### 2.1. The Food Records

FRs are prospective, open-ended survey methods to collect data about food and beverage intake during a certain period. They consist of a self-recording of foods and drinks at the time they are consumed. It is important to train the participants on how to properly report their meals, including information about the location, time, type of food consumed, different ingredients and methods of preparation, portions or weights, and product labels when available. Although a weight record is the most accurate way to correctly quantify the nutritional intake, the portion sizes can be also quantified with the help of a photographic model or standard household measures and then converted by the interviewer in weight [23]. A length of 3 days is the minimum time required to record reliable data on food consumption. Longer times (i.e., 7 days) increase the amount of information and reduce the day-to-day variability, but it has been shown that periods of over four consecutive days are associated with a lower accuracy of the records. Thus, short FRs are more often used and the diary can be filled in non-consecutive days (both weekend and working days) with the aim to collect more representative information of the different dietary habits of the week. The diary is analyzed by trained researchers, also with the possibility of using software programs specifically developed for this purpose, which are equipped with images of food portions and can be used to directly estimate the average daily caloric intake and the percentages of macronutrients [23].

The latest guidelines of the European Association for the Study of the Liver indicate the FRs as the preferred method to assess food intake in patients with cirrhosis, even if in the absence of strong supporting evidence [1].

### 2.2. The 24 h Recall

The 24 h recall (24 hR) is an open-ended retrospective method to investigate dietary intake. It consists of an interview carried out by a trained investigator about all the food and beverages consumed during all the preceding day. It may be computer-assisted and requires the interviewer to be familiar with all the available foods, methods of preparation and the nutritional habits of the population in exam [24].

### 2.3. The Food Frequency Questionnaire

The FFQ is a retrospective, not open-ended method to investigate dietary intake. It allows the assessment of the frequency of consumption of pre-listed foods and beverages during a certain period (usually weeks). Since the items are pre-determined, the choice of the foods and beverages available in the list should be tailored to the population being examined, taking into account the characteristics and the eating habits of the participants. Moreover, the questionnaire should investigate portion sizes to better quantify dietary intake [24].

## 3. Studies Focused on Food Intake in Patients Awaiting LT

We performed an accurate search based on the following Medical Subject Heading (MeSH) keywords: “Food records”, “liver transplantation”, “food diaries”, “caloric intake”, “protein intake”, “liver cirrhosis”. The databases used for the search were: Scopus (www.scopus.com) (1969–2019), MEDLINE (1969–2019), and the US National Library of Medicine (www.PubMed.gov) (to find the ‘in process’ citations missed by MEDLINE). The references of the studies were used to find additional articles. The screening of titles and abstracts was performed by two authors (F.P. and I.B.). Full-length versions of selected articles were then assessed for inclusion criteria: studies in patients aged >18 years; cirrhosis defined on histopathologic, laboratory, clinical and ultrasound features; studies performed on outpatient; paper published in the English language. The following data were collected: number of patients; tool used for the analysis of the nutritional intake; total energy intake; carbohydrates daily intake; protein daily intake; lipids daily intake (Table 3).

It has to be acknowledged that the above cited studies in waitlisted patients were not exclusively focused on the analysis of the dietary intake, which was instead part of a more extensive nutritional assessment often designed to evaluate the changes occurring after LT. Thus, not all the studies reported the daily intake of all macronutrients [9,13,27,28,31] or total calories [13], while, for other studies [9,28,30], we calculated the average daily intakes from the values reported for the different patients’ sub-groups.

The common finding emerging from these studies, which were performed in different areas of the world, is that the majority of patients with cirrhosis awaiting LT presented a state of malnutrition associated with inadequate dietary intakes. In particular, caloric and protein intake was frequently deficient in malnourished patients [9,13,27,28,30,33]. However, even not malnourished patients at the initial assessment had inadequate nutritional intakes that were well below the level of adequacy, in particular regarding caloric and protein intakes [13,28,30,33], but also regarding total carbohydrates and lipids [33]. The cohorts of these studies were heterogeneous and the percentage of patients with inadequate caloric intake ranged from 13.1% (data derived from the weighted average) [28] to 91.8% [30] among different study populations. Moreover, some patients reported an excess of caloric and protein intake [11].

We have recently performed an observational prospective study specifically designed to assess the food intake in a cohort of outpatients with cirrhosis at different stages of severity [34]. The Emilia-Romagna ethic committee (CE-AVEC) reviewed and approved the study (number of approval 33/2014/U/Sper) and all patients gave informed consent before the inclusion. The analysis of the 3d-FR showed unequivocally that these patients have an inadequate, usually deficient, intake of calories and macronutrients, thus presenting poor adherence to the current guidelines even if they received general nutritional recommendations by the attending hepatologist during the normal path of care.

For the purpose of this review, we have repeated the analysis including only the 33 patients who were in the waiting list for LT at the time of the study, including patients with non-advanced hepatocellular carcinoma (HCC) within the Milan criteria. Food intake was calculated by the 3d-FR according to the nutritional tables of products or referring to the Food Composition Database for Epidemiological Studies in Italy [35]. To determine the degree of adherence, the average daily intake of each patient was compared to what is recommended by the latest nutritional guidelines [1]. In case of fluid retention, the dry body weight was estimated in three different ways: (1) referring to a weight registered before the fluid retention, (2) referring to a post-paracentesis body weight or (3) by subtracting a percentage (5, 10 or 15% in relation of the degree of liquid retention) [1]. Each parameter was considered appropriate if it was within the reference range indicated by the nutritional recommendations [1]. Finally, we calculated the degree of inadequacy in terms of distance from the reference range.

Patients were predominantly male (86%), with a median age of 57.1 ± 5.99 years and a mean BMI of 27.5 ± 4.5. Nine patients (27%) were in Child-Pugh class A, 20 (61%) in class B and four (12%) in class C. HCC was present in 14 patients (42%). As expected, prognostic scores were higher in patients without HCC as compared to those with HCC (Child-Pugh: 8.6 ± 1.8 vs. 6.8 ± 1.5; Model for End stage Liver Disease (MELD): 17.4 ± 2.7 vs. 11.6 ± 3.5).

As shown in Table 4, the analysis of the 3d-FR has demonstrated a level of adequacy equal to 58% and 61% for the daily consumption of lipids and simple carbohydrates, respectively. Instead, a completely different picture has emerged regarding the intakes of calories, protein, and complex carbohydrates, with a clear majority of patients presenting an insufficient intake (79–94%).

We also analyzed the number and distribution of meals, considering both main meals and snacks. Only 17 patients (52%) had at least four meals per day, which was considered the threshold to define the adequate the number of daily meals. Moreover, a late-night snack was eaten by 11 out the 17 patients (65%) having at least four meals per day. In contrast, none of the 16 patients without a sufficient number of daily meals declared eating a late-night snack. Finally, only 27% of patients self-reported any physical activity.

No statistically significant difference emerged among patients grouped according the Child–Pugh classes in terms of inadequacy of macronutrients intake and eating patterns, likely as a result of the low sample size, particularly in the Child-Pugh C group.

**Figure 1:**
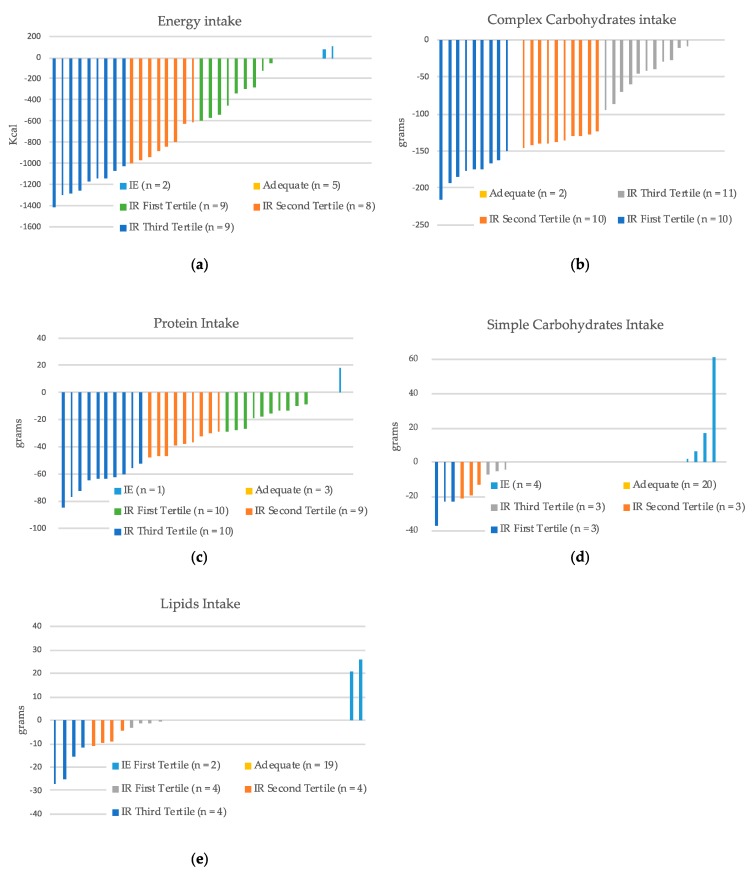
Degree of inadequacy for each macronutrient intake from the normal ranges indicated by international guidelines. The distribution of the degree of inadequacy from the range of normal intake is shown in the tertiles (*n* = 33). (**a**) Energy intake; (**b**) Complex carbohydrates intake; (**c**) Protein intake; (**d**) Simple carbohydrates intake; (**e**) Lipids intake. IR: inadequate reduced. IE: inadequate in excess.

As shown in Figure 1, when the study population was divided according to the tertiles of inadequacy, the distribution of patients appeared to be homogeneous among the tertiles. Moreover, a considerable number of patients had a nutritional intake far from the level of adequacy. As an example, over 50% of patients with insufficient caloric intake presented a deficit higher than 800 kcal/day.

Finally, it is worth of mentioning that waitlisted patients presented a much higher willingness to complete the FRs as compared to those observed in patients with cirrhosis not listed for LT (33/40 (82.5%) vs. 45/121 (37.2%)). In summary, our data are consistent with the findings of the studies mentioned above, in particular regarding the inadequacy of nutritional intake.

## 4. Discussion

The major finding emerging from the studies assessing the food intake in patients waiting for LT is represented by the low-level adherence to the nutritional recommendations provided by the current international guidelines [1]. This result raises two important questions.

The first question is: why is the diet of these patients so far from what is recommended? There are several reasons to explain this finding: poor knowledge of patients and physicians on the composition of food and drinks, family environment and low socio-economic conditions, low consciousness of the key role of nutrition for the management of the disease that leads to general poor compliance with prescribed diets, or not enough time to provide nutritional recommendation in the daily clinical practice [2,13]. Interestingly, the common wisdom that “fat and sweet” food consumption is a damaging habit may have contributed to the higher adherence observed for lipid and simple carbohydrate intake.

The second question is: how can we improve the food intake of these patients? In most cases according to the regular planned path of care, the physician provides patients with general advice on liquid and food consumption with the support of simple written material. The results available highlight that this approach is not sufficient and there is a need to implement an educational program provided by certified nutritionists or dieticians. The high motivation usually observed in patients waiting for LT may also favor their compliance to a nutritional program. Interestingly, in our analysis, the proportion of patients accepting the nutritional evaluation was significantly higher in waitlisted patients as compared to candidates for LT.

Despite the possibility that HCC could have been a confounding factor in our study, in the 14 patients with non-advanced HCC, the burden of active tumor was quite limited and they presented better liver function compared to patients without HCC.

A close collaboration between hepatologists and nutritionists is warranted since the nutritional requirements of patients with end-stage cirrhosis can greatly change due to many complications of the disease, such as ascites, hepatic encephalopathy, renal failure, and diabetes. In fact, some of the interventions needed to manage these complications can have a negative impact on the nutritional balance of these patients [36]. As examples, sarcopenia can be worsened by the low-protein diet prescribed to prevent the recurrence of hepatic encephalopathy [5], or the adoption of a low-sodium diet in order to manage ascites can make food less palatable, leading to a further reduction in caloric intake [36]. Thus, the nutritional intervention should be tailored to the specific needs of the individual patient.

## 5. Conclusions

Patients with cirrhosis waiting for LT present a very low adherence to recommendations provided by the international nutritional guidelines. Due to the poor prognostic impact of nutritional disorders on mortality and morbidities before and after LT, the assessment of the daily food intake, within the frame of a global evaluation of nutritional status, should be part of the management of these patients. Moreover, any nutritional intervention should be the result of a multidisciplinary approach involving both hepatologists and nutritionists and tailored to the specific needs of each patient.

## Figures and Tables

**Table 1:** General nutritional recommendations for patients with cirrhosis.

Nutritional Recommendations	
Energy	35 kcal/kg body weight ^1^
Protein	1.2–1.5 g/kg body weight ^2^
Total Carbohydrates	45–75% of caloric intake
Simple Carbohydrates	10–15% of caloric intake
Fibers	25–45 g/daily
Lipids	20–30% of caloric intake
**Special Considerations**	
Hepatic Encephalopathy	Increase BCAAs and decrease ammonia intake
Ascites	Fluid restriction and low-sodium intake (<2 g/day)

^1^ Actual body weight if BMI < 25, actual body weight—500/850 kcal if BMI ≥ 25, dry body weight in patients presenting fluid retention [1]. ^2^ Actual body weight if BMI < 25, ideal body weight if BMI ≥ 25, dry body weight in patients presenting fluid retention [1]. Abbreviations: BCAA: branched chain amino acid, BMI: body mass index.

**Table 2:** Main advantages and disadvantages of methods to assess food intake.

	Advantages	Disadvantages
**Food Records**	Detailed information on the actual food intake at the time of recording. Absence of recall bias since the diary should be filled in at the time of food consumption. Interviewer not required to collect information.	All participants need to be highly motivated and literate to properly fill in all the fields of the diary, thus excluding some groups of patients or limiting the accuracy of their report. In order to overcome this limitation, some devices, including voice records, food atlas, camera and mobile phones, can be also used. Time-consuming method. Participants could modify their dietary intake during their recording days.
**24 h Recall**	Short time required. Applicable in large studies including patients with different ethnicity. No literacy required. No influence on the eating habits.	Relies on the memory of the participants. Portion sizes can be difficult to be quantified. Trained interviewers are required. A single test may not be representative of the real patient’s eating habit. Thus, to account for day-to-day variations, the 24 h recall (24 hR) should be repeated several times [1].
**FFQ**	Relatively low-cost method. Short time required, especially when self-administered. Easier data management since in most cases it is pre-coded. A large population can be investigated.	It relies on the long-term memory of the participants, referring to several weeks. Quantification of portions could be inaccurate. It is not an open-ended method, so some foods could not be reported if they are not in the list of available items.

**Table 3:** Analysis of the nutritional intake in patients with cirrhosis waiting for liver transplantation in published studies.

Authors	*n*	Tool	Total Energy Intake (kcal/Day)	Carbohydrates (g)	Protein (g)	Lipids (g)
Ferreira et al. [25]	17	3d-FR	1670.5 ± 489.8 *	236.5	72.9	48.2
Lunati et al. [12]	84	3d-FR	2006 ± 624 *	285.9	75.2	62.4
Brito-Costa et al. [26]	56	24 hR	2062.8 ± 797.8 *	259.4	94.9	71.5
Mc Coy et al. [11]	17	7d-FR	2257.2 ± 605.9 *	281	132.6	73
Merli et al. [27]	25	‡	2030 (1610–2870) ^†^	−	63	−
Marr et al. [28]	70	3d-FR	1766.4 *	−	−	−
Andrade et al. [29]	23	24 hR	1774.3 ± 537.9 *	234.5	93.6	53.9
Ney et al. [13]	630	2d-FR + FFQ	−	−	68.8	−
Ferreira et al. [30]	73	3d-FR	1485.1 (559.3–3432) ^†^	218.1	60.5	42.8
Merli et al. [9]	38	‡	2006 ± 423 *	−	−	−
Ferreira et al. [31]	16	3d-FR	1520 (576–2713.6) ^†^	−	−	−
Richardson et al. [32]	23	3d-FR	1542 ± 124 *	199	60.3	62.3
Ferreira et al. [33]	159	24 hR	1490.9 ± 580.7 *	225.7	56	36.7

Abbreviations: 3d-FR: three-days food records; 24 hR: twenty-four hour recall; 7d-FR: seven-days food records; FFQ: food frequency questionnaire; 2d-FR: two-days food records; − not found in the article; * the mean ± standard deviation. ^†^ the median range. ‡ interviews and analysis performed with the use of specific software (WinFood, Medimatica, Colonnella Teramo, Italy).

**Table 4:** Patient adherence to nutritional intake according to international nutritional guidelines (*n* = 33).

Energy and Macronutrients Intake	Adequate	Inadequate Reduced	Inadequate Excessive
Total energy intake	5 (15%)	26 (79%)	2 (6%)
Protein intake	3 (9%)	29 (88%)	1 (3%)
Complex carbohydrates intake	2 (6%)	31 (94%)	0 (0%)
Lipids intake	19 (58%)	12 (36%)	2 (6%)
Simple carbohydrate intake	20 (61%)	9 (27%)	4 (12%)

Each parameters was considered appropriate if included within the reference range indicated by the guidelines [1].
